# Head to head comparison between arterial spin labelling MRI and [^18^F]FDG-PET in presurgical evaluation of epilepsy in children: the role of voxel-based asymmetry index analysis

**DOI:** 10.1007/s10072-026-08891-y

**Published:** 2026-02-27

**Authors:** Pietro Mattioli, Giulia Nobile, Matteo Cataldi, Luca Bosisio, Francesco Famà, Andrea Rossi, Alessandro Consales, Stefano Raffa, Flavio Villani, Silvia Morbelli, Dario Arnaldi, Lino Nobili, Domenico Tortora, SL&EPgrg, Alessandra Ferrari, Stefano Francione, Thea Giacomini, Laura Giorgetti, Mattia Losa, Maria Margherita Mancardi, Valentina Marazzotta, Elisa Micalizzi, Mattia Pacetti, Irene Pappalardo, Costanza Parodi, Giulia Prato, Mariasavina Severino, Laura Siri, Andrea Michele Wolfler

**Affiliations:** 1 Department of Neurology, Rehabilitation, Ophthalmology, Genetics, Maternal and Child Health (DINOGMI), University of Genoa, Genoa, Italy; 2 Division of Clinical Neurophysiology and Epilepsy Centre, IRCCS Ospedale Policlinico San Martino, Genoa, Italy; 3 Child Neuropsychiatry Unit, IRCCS Istituto Giannina Gaslini, European Reference Network EpiCARE, Genoa, Italy; 4 Neuroradiology Unit, IRCCS Istituto Giannina Gaslini, Genoa, Italy; 5 Department of Health Sciences (DISSAL), University of Genoa, Genoa, Italy; 6 Division of Neurosurgery, IRCCS Istituto Giannina Gaslini, Genoa, Italy; 7 Nuclear Medicine Unit, IRCCS Ospedale Policlinico San Martino, Genoa, Italy; 8 Nuclear Medicine Unit, University of Turin, Turin, Italy

**Keywords:** Arterial spin labelling (ASL) MRI, [^18^F]FDG-PET, Children with epilepsy, Epilepsy surgery

## Abstract

**Background and purpose:**

Arterial Spin Labelling MRI is a neuroimaging technique able to evaluate brain perfusion, an indirect measure of brain metabolism and function. Arterial Spin Labelling MRI showed to have performances comparable to [^18^F]fluorodeoxyglucose-PET in in epilepsy, yet literature data is still lacking about its use in children and the value of voxel-based asymmetry index analysis. Purpose of the project is to compare the Arterial Spin Labelling MRI and [^18^F]fluorodeoxyglucose-PET ability to identify the epileptogenic zone before and after asymmetry index analysis in children.

**Materials and methods:**

In this observational study, paediatric patients with focal onset drug-resistant epilepsy that underwent presurgical evaluation, including Arterial Spin Labelling MRI and [^18^F]fluorodeoxyglucose-PET, were enrolled. The epileptogenic zone was defined by anatomo-electroclinical correlation and post-surgical outcome, when feasible. The rates of concordance with the epileptogenic zone of Arterial Spin Labelling MRI and [^18^F]fluorodeoxyglucose-PET before (visual analysis) and after asymmetry index analysis, were calculated. Statistically significant differences between were determined using Mc Nemar’s test (*p* < 0.05).

**Results:**

28 paediatric patients (mean age 10.07 years, 15 females) with focal epilepsy were enrolled; 22 underwent epilepsy surgery (mean age 9.86 years, 12 females). When comparing the techniques, visual analysis of Arterial Spin Labelling MRI had a significantly lower rate of concordance with the epileptogenic zone (*p* < 0.05).

**Conclusion:**

Voxel-based asymmetry index analysis increased significantly the rate of concordance of Arterial Spin Labelling MRI with the epileptogenic zone, achieving results comparable with [^18^F]fluorodeoxyglucose-PET in a cohort of paediatric patients.

**Supplementary Information:**

The online version contains supplementary material available at 10.1007/s10072-026-08891-y.

## Introduction

Epilepsy is one of the most prevalent neurological disorders [1]. Despite advancements in pharmacological treatments, approximately one-third of individuals with epilepsy continue to suffer from seizures despite optimal medical management. This subgroup of patients experiences substantial morbidity and a reduced quality of life [2, 3]. This is especially important for paediatric patients, who have a longer life expectancy, and an early and effective intervention is crucial, as prolonged seizure activity can lead to adverse neurodevelopmental outcomes [4]. For these patients, surgical intervention offers a potential remedy, particularly when the epileptogenic zone (EZ), the area of the brain responsible for seizure generation, can be precisely identified and resected.

To achieve accurate localization, a multimodal approach often combines both non-invasive and invasive techniques. Among these modalities, [^18^F]fluorodeoxyglucose PET ([^18^F]FDG-PET) has the ability to measure metabolic activity in the brain [5]. However, even if it uses very low doses of ionizing radiations, [^18^F]FDG-PET is considered a minimally invasive procedure and, in paediatric pre-surgical program, it often requires a further general anaesthesia. For such reasons, arterial spin labelling MRI (ASL) has emerged as a promising tool for the non-invasive assessment of cerebral perfusion [6–9]. Indeed, ASL uses magnetically labelled arterial blood water as an endogenous tracer to measure CBF. This method provides quantitative maps of CBF without the need for exogenous contrast agents or ionizing radiation, making it especially appealing for use in children [10]. In the context of epilepsy, altered perfusion patterns may correlate with the EZ, indicating abnormal neural activity [11]. Despite its potential, the application of ASL in identifying the EZ has been predominantly explored in adults with temporal lobe epilepsy [9, 12], and there have been only few researches conducted in paediatric populations [13, 14]. Furthermore, there is variability in the approach employed to compare ASL and [^18^F]FDG-PET [7, 15, 16], and of the way of applying off-station analysis, such as voxel-based asymmetry index analysis (A-Index) [17].

The purpose of this study was to compare the ability of ASL and [^18^F]FDG-PET, both before (visual analysis) and after (asymmetry index analysis) A-Index, to identify the EZ in children undergoing presurgical evaluation for drug-resistant focal epilepsy. We hypothesized that A-Index enhances the detection of subtle asymmetries in imaging data, which can be particularly useful in identifying the EZ in cases where traditional visual inspection may fall short. By employing this comparative approach, we aimed to determine whether ASL and [^18^F]FDG-PET have comparable abilities in localizing the EZ and to identify the role of asymmetry index analysis.

## Materials and methods

All patients with unilateral focal epilepsy that underwent presurgical evaluation for epilepsy, including brain [^18^F]FDG-PET and MR perfusion study were enrolled. Patients with motion artifact at MR perfusion study were excluded. Demographic and clinical features were collected for every enrolled patient. Each patient underwent at least video-EEG, 3 T brain MRI, and [^18^F]FDG-PET in accordance with good clinical practice for presurgical evaluation for epilepsy surgery.

The EZ was determined by the analysis of the anatomo-electro-clinical correlations performed by the multidisciplinary team meeting, and, when feasible, the presence of seizure freedom at post-surgical follow up.

### Magnetic resonance imaging acquisition

MRI was performed according to the harmonized neuroimaging of epilepsy structural sequences—HARNESS-MRI protocol [18]. Also, all patients underwent an MRI perfusion study with background-suppressed three-dimensional dimension Pseudo-continuous Arterial Spin Labeling as described in a previous paper from our group [19] and detailed in current guidelines [20].

### [^18^F]fluorodeoxyglucose positron emitting tomography

Brain [^18^F]FDG-PET was performed in accordance with the European Association of Nuclear Medicine guidelines [21]. Subjects fasted for at least six hours. Before the radiopharmaceutical injection, blood glucose was checked and was < 7.8 mmol/L in all cases. After a 10-minute rest in a silent and obscured room with eyes open and ears unplugged, subjects were injected with approximately 200 MBq of [^18^F]FDG via a venous cannula. They remained in the room for 30 min after the injection and then moved to the PET room, where scanning started approximately 45 min after the injection and lasted 15 min. A polycarbonate head holder was used to reduce head movements during the scan. Images were acquired by means of SIEMENS Biograph 16 PET/CT equipment with a total axial field of view of 15 cm and no interplane gap space. The attenuation correction was based on CT. Images were reconstructed through an ordered subset expectation maximization algorithm, 16 subsets, and 6 iterations, with a reconstructed voxel size of 1.33 × 1.33 × 2.00 mm.

### Neuroimaging analysis and post-processing

Firstly, ASL and [^18^F]FDG-PET, after co-registration in the 3D-T1 high-resolution space, were visually evaluated by an expert neuroradiologist and an expert nuclear medicine physician.

Visual analysis of ASL was performed after defining a standard method of windowing of CBF maps, setting the highest values of the visual windowing within the range of 80–90 ml/100 g/min, and the lowest value at 0 ml/100 g/min. For visual analysis of [^18^F]FDG-PET, auto-windowing was a priori based on the striatum considered as a reference region with the greatest [^18^F]FDG uptake in normal scans.

Hence, a region of interest for altered CBF and for altered brain metabolism were visually identified (ASL visual analysis (V-ASL) and [^18^F]FDG-PET visual analysis (V-PET), respectively).

Subsequently, A-Index was calculated for ASL and [^18^F]FDG-PET according to the method described by Boscolo Galazzo et al. [6] Briefly, ASL and [^18^F]FDG-PET maps in the native space were affine-registered to the individual 3D-T1 high-resolution anatomical images by using the FLIRT tool of FSL. Each T1-weighted image was then registered to the Montreal Neurological Institute (MNI) space with 1mm3 resolution using a non-linear method (FNIRT tool in FSL). Finally, transformation parameters were combined to spatially normalize the ASL and [^18^F]FDG-PET maps in the MNI space. The registered maps were then smoothed with a 2 × 2 × 2 mm FWHM Gaussian kernel. Then, a voxel-wise asymmetry index calculation was performed using the following formula: A-index = 100 × (Right − Left)/(Right + Left). An expert neuroradiologist and an expert nuclear medicine physician evaluated the obtained maps, coregistered to the individual 3D-T1 high-resolution anatomical images, to identify the clusters of major asymmetry between right and left hemispheres for both techniques (ASL voxel-based asymmetry index analysis, (A-Index-ASL) and [^18^F]FDG-PET voxel-based asymmetry index analysis, (A-Index-PET)).

Regions of altered perfusion/metabolism (V-ASL/V-PET) and clusters of asymmetry (A-Index-ASL/A-Index-PET) were classified at a sublobar level, as previously described for other neuroimaging techniques [22]. Of notice, the observers were allowed to identify more than one region/cluster, as it is expected in the clinical practice, and were blind to the surgical outcome and the other neuroimaging technique, but not to the EEG and the structural MRI data.

For each patient, a degree of concordance of V-ASL/V-PET and the A-Index-ASL/A-Index-PET with the EZ, was determined. The degree of concordance with the EZ was defined at sublobar level and as follows:

I. no concordance (no overlap between regions/clusters and the EZ),
II. partial concordance (the EZ is included between the regions/clusters identified, but either one region/cluster is not part of the EZ, or part of the EZ is not included in the regions/clusters identified),
III. complete concordance (complete overlap between regions/clusters and the EZ, at sublobar level).

**Fig. 1:**
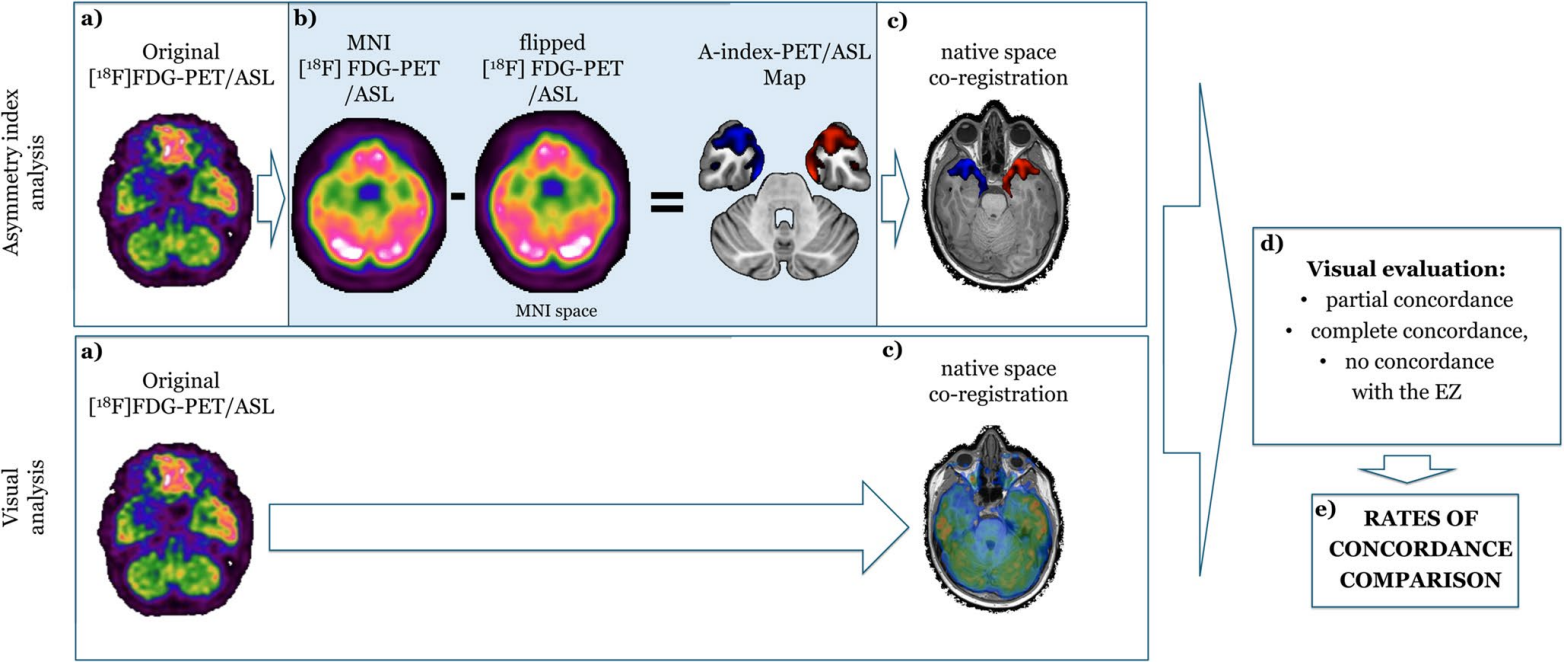
Workflow of analysis. The workflow of the performed analysis is briefly documented. In the top row, the asymmetry index analysis is reported: after acquisition of the raw [^18^F]FDG-PET/ASL-MRI image (**a**), asymmetry index analysis is performed to obtain an A-Index-PET/A-Index-ASL map (**b**), that is visually evaluated after co-registration in the 3D-T1 native space (**c**). In the bottom row, the visual analysis (V-PET/V-ASL) is reported: the original data is directly co-registered in the 3D-T1 native space (**c**). Finally, resulted images are visually evaluated to attribute a rate of concordance (partial, complete, no concordance) with the EZ (**d**) to be compared between each other (**e**)

Neuroimaging analysis and comparisons are schematically represented in Fig. 1.

### Statistical analysis

As a first descriptive analysis, clinical, instrumental data and the outcomes of enrolled patients were explored. Then, the performances of V-ASL, A-Index-ASL, V-PET and A-Index-PET were compared.

The proportions of concordance of the neuroimaging technique were compared before and after dichotomization of the degree of concordance into two classes: “concordance” and “no concordance” by merging the “partial” and “complete” concordance classes. We considered such dichotomization important from the clinical perspective in the setting of a multimodal neuroimaging approach to presurgical evaluation for epilepsy.

Since neuroimaging technique and post-processing were dependent, categorical variables, Mc Nemar-Bowker test for marginal homogeneity or Mc Nemar test with continuity correction (dichotomous variables) was used to compare the proportion of concordance with the EZ.

The same analysis was repeated in the subgroup of patients in which the EZ defined by anatomo-electroclinical correlation was removed and the patients resulted to be seizure free after a follow up of at least 12 months (true EZ).

The statistical analysis was performed in “r” implemented in BlueSky statistics, p-values below 0.05 were considered statistically significant.

## Results

Twenty-eight patients (15 females, 10.07 ± 4.6 years) that underwent presurgical evaluation for epilepsy were enrolled. Twenty-two patients underwent epilepsy surgery, of these 17 had the EZ completely removed, and 14 were seizure free at follow up. Of notice, in one patient the EZ was not completely removed, but the outcome resulted in seizure freedom at follow up. Therefore, 13 out of 17 patients (76.47%) in which the EZ was considered as completely removed were free from seizures at follow up.

Clinical, instrumental and outcome data of the whole group and the subgroup of patients that underwent epilepsy surgery are reported in Tables 1 and 2, respectively. Notably, 14 patients (66.7%) had an etiological diagnosis of focal cortical dysplasia (FCD), whereas the remaining patients showed heterogeneous etiologies, including one case of non-specific gliosis, one dysembryoplastic neuroepithelial tumor, one glioneuronal tumor, two low-grade gliomas, two cases of hyaline protoplasmic astrocytopathy, and one case with unknown etiology at the time of manuscript preparation.

**Table 1:** Patients demographic, clinical and instrumental features. Data are reported as average and standard deviation (in brackets) for continuous variables or absolute number and percentage (in brackets) for categorical variables

	Overall (*N* = 28)
Age (at ASL, years)	10.07 (4.64)
Sex (females)	15 (53.6%)
Age of seizures’ onset	5.39 (4.771)
Epilepsy surgery (performed)	22 (78.6%)
Months between ASL and [^18^F]FDG-PET	7.68 (10.85)
Extra temporal lobe EZ (presence)	18 (64.29%)
Sedation during ASL (presence)	13 (46.4%)
Sedation during [^18^F]FDG-PET (presence)	13 (46.4%)
Seizures 48 h before ASL (presence)	8 (28.6%)
Seizures 48 h before [^18^F]FDG-PET (presence)	9 (32.1%)

Legend: ASL: arterial spin labelling MRI; EZ; epileptogenic zone; [^18^F]FDG-PET: [^18^F]fluoro-deoxiglucose PET

**Table 2:** Demographic, clinical and instrumental features of patients that underwent epilepsy surgery. Data are reported as average and standard deviation (in brackets) for continuous variables or absolute number and percentage (in brackets) for categorical variables

	Overall (*N* = 22)
Age (at ASL, years)	9.87 (4.76)
Sex (females)	12 (54.5%)
Age of seizures’ onset	5.000 (4.46)
Follow up time (months)	29.14 (14.23)
Engel	
- Ia	14 (63.6%)
- IIa	2 (9.1%)
- IIb	2 (9.1%)
- IIIa - IV	4 (18.2%) 0 (0%)
Seizure freedom (presence)	14 (63.6%)
Focal cortical dysplasia (presence)	14 (66.7%)
Gross EZ resection (presence)	16 (72.7%)
Months between ASL and [^18^F]FDG-PET	7.00 (11.37)
Sedation during ASL (presence)	11 (50.0%)
Seizures 48 h before ASL (presence)	6 (27.3%)
Sedation during [^18^F]FDG-PET (presence)	11 (50.0%)
Seizures 48 h before [^18^F]FDG-PET (presence)	7 (31.8%)

Legend: ASL: arterial spin labelling MRI; EZ; epileptogenic zone; [^18^F]FDG-PET: [^18^F]fluorodeoxiglucose PET

When comparing proportion of concordance of each neuroimaging techniques with the EZ, no significant differences were found between A-Index-ASL, V-PET and A-Index-PET, whereas V-ASL showed a significant lower rate of concordance, when compared with A-Index-ASL, V-PET, and A-Index-PET (Table 3). An explanatory example of one case is documented in Supplementary Fig. 1.

**Table 3:** Rates of agreement with the epileptic zone (anatomo-electroclinical-correlation) through neuroimaging techniques before and after voxel-based asymmetry index post-processing. Significant results are reported in **bold** (p values)

	Neuroimaging concordance proportion				Comparisons					
	V-ASL	A-Index-ASL	V-PET	A-Index-PET	V-ASL vs. A-Index-ASL	V-ASL vs. A-Index-PET	V-ASL vs. V-PET	A-Index-ASL vs. V-PET	A-Index-ASL vs. AI-PET	V-PET vs. A-Index-PET
**Agreement** ^*^					**0.005**	**0.012**	**0.005**	0.842	0.584	0.485
No concordance	16 (57.14%)	2 (7.14%)	3 (10.71%)	4 (14.29%)						
Partial concordance	5 (17.86%)	9 (32.14%)	11 (39.29%)	11 (39.29%)						
Complete concordance	7 (25.00%)	17 (60.71%)	14 (50.00%)	13 (46.43%)						
**Dichotomized** ^#^					**0.001**	**0.006**	**0.001**	0.9	0.683	0.9
No concordance	16 (57.14%)	2 (7.14%)	3 (10.71%)	4 (14.29%)						
Concordance	12 (42.86%)	26 (92.86%)	25 (89.29%)	24 (85.71%)						

Legend: A-Index-ASL: arterial spin labelling MRI voxel-based asymmetry index analysis; A-Index-PET: [^18^F]fluorodeoxyglucose PET voxel-based asymmetry index analysis; V-ASL: arterial spin labelling MRI visual analysis; V-PET: [^18^F]fluoro-deoxiglucose-PET visual analysis

*Mc Nemar-Bowker for marginal homogenety

#Mc Nemar test with continuity correction

Finally, when considering the subgroup of 13 patients in which the EZ was considered as completely removed and that were seizure free at follow up, the V-ASL was confirmed to have a lower rate of agreement with the “true” EZ as compared with A-Index-ASL, V-PET and A-Index-PET, without reaching statistical significance, likely because of the smaller sample size (Supplementary Table 1).

## Discussion

In this study we compared the rate of concordance of [^18^F]FDG-PET and ASL before and after the application of asymmetry index analysis with the epileptogenic zone in a cohort of paediatric patients affected by focal epilepsy who underwent presurgical evaluation at a tertiary epilepsy centre. We found that after voxel based asymmetry index analysis, ASL result achieved a concordance with the EZ comparable to that of [^18^F]FDG-PET.

An increased or a decreased function of a brain region is usually coupled with a higher or lower brain perfusion and metabolism [11]. Indeed, the function of a brain region directly depends on glucose metabolism and therefore on its perfusion [6]. Hence, it is expected from neuroimaging techniques that evaluate brain perfusion, such as ASL, or brain metabolism, such as [^18^F]FDG-PET, to return similar results. In the field of focal epilepsy, literature about the performance of ASL is growing, but data on extratemporal epilepsy, paediatric patients and the effect of post-processing are still lacking [17, 19, 23, 24]. In particular Khalaf and colleagues found that ASL and [^18^F]FDG-PET had substantial agreement in a cohort of adult patients and that the combination of ASL with [^18^F]FDG-PET may increase sensitivity and specificity so to detect the epileptogenic zone in temporal lobe epilepsy [13]. Shang et al. on the other hand found that ASL and [^18^F]FDG-PET after asymmetry index analysis did not differ significantly in their performances in detecting the EZ in a group of adult patients with temporal and extratemporal epilepsy [25]. Moreover, there is increasing evidence that the integration of ASL within the presurgical evaluation for epilepsy in the multimodal scene may increase the diagnostic accuracy [26]. Finally, ASL performs well even in children with poorly defined focal epilepsy, showing a concordance with [^18^F]FDG-PET in 75% of cases [14]. In their project, Lam and colleagues evaluated the ASL visually and after post-processing, by applying a more strict, yet elegant, classification of the asymmetry index, based on z-scores and cluster cutoff percentage (of 1.5 and 5%, respectively) [14]. In this study, we confirmed these findings in an independent cohort of paediatric patients, employing a more subjective approach. While we acknowledge that the absence of a strict classification by z-scores and cluster size introduces potential biases due to the final subjective decision, our aim was to allow for some level of interpretation by the observer, thereby better reflecting clinical practice.

Nonetheless, several post-processing techniques were applied to ASL [8, 15]. Among these, voxel based asymmetry index analysis showed promising results [6, 16, 19, 23, 27]. In our study we found a significant effect of A-Index on ASL, in agreement with the literature [6, 8]. Interestingly, however, the application of A-Index did not have significant effects on [^18^F]FDG-PET. This is an unexpected finding, since it has been previously described how A-Index increases the diagnostic accuracy of visual analysis of [^18^F]FDG-PET [28, 29]. The literature on the topic, however, is still lacking and needs further research [30]. A possible explanation may reside in the classification method that we used, that is the agreement with the EZ either in a dichotomic or semi quantitative classification. This required the rater to assign a specific score, which inevitably simplified the original imaging report but was necessary to enable statistical analysis. Such an approach may have underweighted multifocal or extra-EZ (widespread/remote) abnormalities described in the original report, potentially inflating the estimated concordance with the EZ. Moreover, to mimic the clinical practice, we provided to the raters the EEG and morphological MRI data of the patients, that probably increased their ability to visually interpret the neuroimaging. Therefore, it is possible that the very good performance of V-PET is related to a roof effect that hid the benefits of voxel-based asymmetry index. Even if this may be considered as an interpretative bias, the same effect was not detectable in V-ASL that had performances significantly lower, underlying the importance of post-processing in this specific neuroimaging technique.

The EZ has been commonly defined as the region of the brain whose removal leads to a complete seizure freedom and, even with stereo-EEG is difficult to determine without an adequate post-surgical follow up [31]. For this reason, to increase the statistical significance of the study, we decided to evaluate the performances of V-ASL, A-Index-ASL, V-PET, A-Index-FDG also in the subgroup of patients undergoing epilepsy surgery, with the EZ being removed, and with a follow up time longer than 12 months. The results of such analysis were similar to the whole group analysis but without reaching a statistical significance, likely because of the smaller sample size. Nevertheless, V-ASL was confirmed to have a tendency to a lower agreement with the EZ as compared to V-PET, A-Index-PET and particularly, A-Index-ASL.

This study has some limitations, that need some discussion. Firstly, patients’ heterogeneity, having one third of them an aetiology different than focal cortical dysplasia, while 63.6% achieved seizure freedom, however, such heterogeneity reflect the design of the study, that is observational and retrospective. Moreover, the most homogenous subgroup of 13 patients with a complete removal of the EZ obtained similar, albeit not significant, results, advocating the need for further, larger studies. Secondly, the sample size is relatively small, yet, to our knowledge, very few studies compared the concordance of ASL and [^18^F]-FDG-PET in the paediatric population, being the study of Lam and colleagues [14], the largest. Present study is not the first study comparing ASL-MRI with other neuroimaging techniques, indeed, in a very similar fashion, Lam and colleagues evaluated the power of ASL-MRI to identify the EZ and compared such results with other neuroimaging techniques, including [^18^F]-FDG-PET. The results of our study underline that the concordance of ASL perfusion abnormalities with the epileptogenic zone is comparable to that of [^18^F]-FDG-PET when the A-index is applied, suggesting ASL as a potential alternative to brain metabolism assessment in pediatric patients. Moreover, MRI and [^18^F]-FDG-PET were not acquired simultaneously, and in four cases more than one year apart. This is a possible bias in the interpretation of the results since the functional networks in paediatric patients can change relatively quickly and being drug-resistant patients, the different antiseizure medications may have influenced the results. However, such bias does not impact the comparison of the techniques before and after asymmetry analysis. Finally, sedation was necessary in a half of the patient. The effect of sedation is a well-known modifier of brain metabolism [32], hence it may have affected the results of brain [^18^F]-FDG-PET (and probably of ASL-MRI), however, the very same patients underwent sedation in both techniques and the effect of the sedation did not affect the comparison of the same technique before and after A-Index.

## Conclusion

Asymmetry index analysis increased the concordance of ASL results with the EZ, achieving performance levels comparable to those of [^18^F]FDG-PET. In our view, ASL-MRI cannot yet replace [^18^F]FDG-PET, as its interpretation relies heavily on the use of “off-station” analyses, such as A-Index. Nonetheless, if the findings of this and previous studies are validated through larger multicentre projects, ASL could potentially emerge as a non-invasive alternative to [^18^F]FDG-PET in the future. This would offer several advantages for children, including eliminating exposure to ionizing radiation and reducing the number of diagnostic tests required during presurgical evaluations.

## Supplementary Information

Below is the link to the electronic supplementary material.

Supplementary File 1 (DOCX 748 KB)

Supplementary File 2 (DOCX 40.0 KB)

## Data Availability

Data used for the analysis can be requested upon reasonable request to the corresponding author.
