# Correction to: Head to head comparison between arterial spin labelling MRI and [18F]FDG-PET in presurgical evaluation of epilepsy in children: the role of voxel-based asymmetry index analysis

**DOI:** 10.1007/s10072-026-08950-4

**Published:** 2026-03-26

**Authors:** Pietro Mattioli, Giulia Nobile, Matteo Cataldi, Luca Bosisio, Francesco Famà, Andrea Rossi, Alessandro Consales, Stefano Raffa, Flavio Villani, Silvia Morbelli, Dario Arnaldi, Lino Nobili, Domenico Tortora, SL&EPgrg, Alessandra, Ferrari Stefano Francione, Thea Giacomini, Laura Giorgetti, Mattia Losa, Maria Margherita Margherita, Valentina Marazzotta, Elisa Micalizzi, Mattia Pacetti, Irene Pappalardo, Costanza Parodi, Giulia Prato, Mariasavina Severino, Laura Siri, Andrea Michele Wolfler

**Affiliations:** 1 Department of Neurology, Rehabilitation, Ophthalmology, Genetics, Maternal and Child Health (DINOGMI), University of Genoa, Genoa, Italy; 2 Division of Clinical Neurophysiology and Epilepsy Centre, IRCCS Ospedale Policlinico San Martino, Genoa, Italy; 3 Child Neuropsychiatry Unit, IRCCS Istituto Giannina Gaslini, European Reference Network EpiCARE, Genoa, Italy; 4 Neuroradiology Unit, IRCCS Istituto Giannina Gaslini, Genoa, Italy; 5 Department of Health Sciences (DISSAL), University of Genoa, Genoa, Italy; 6 Division of Neurosurgery, IRCCS Istituto Giannina Gaslini, Genoa, Italy; 7 Nuclear Medicine Unit, IRCCS Ospedale Policlinico San Martino, Genoa, Italy; 8 Nuclear Medicine Unit, University of Turin, Turin, Italy

**Correction to: Neurological Sciences**

10.1007/s10072-026-08891-y

In the original version of the manuscript, the institutional author names were listed as main authors. These members should be listed as collaborators, indexed online, and should not be listed as main authors.

The correct list of authors for this paper is shown below:

Pietro Mattioli

Giulia Nobile

Matteo Cataldi

Luca Bosisio

Francesco Famà

Andrea Rossi

Alessandro Consales

Stefano Raffa

Flavio Villani

Silvia Morbelli

Dario Arnaldi

Lino Nobili

Domenico Tortora

SL&EPgrg

The original article has been corrected.

