# Supplementary material for: Head to head comparison between arterial spin labelling MRI and [18F]FDG-PET in presurgical evaluation of epilepsy in children: the role of voxel-based asymmetry index analysis
Source: Neurol Sci. 2026 Feb 27;47(3):302. doi: 10.1007/s10072-026-08891-y (PMC12948786; doi:10.1007/s10072-026-08891-y)
Supplement: Supplementary file 1 — (DOCX 748 KB) [file 10072_2026_8891_MOESM1_ESM.docx]

**
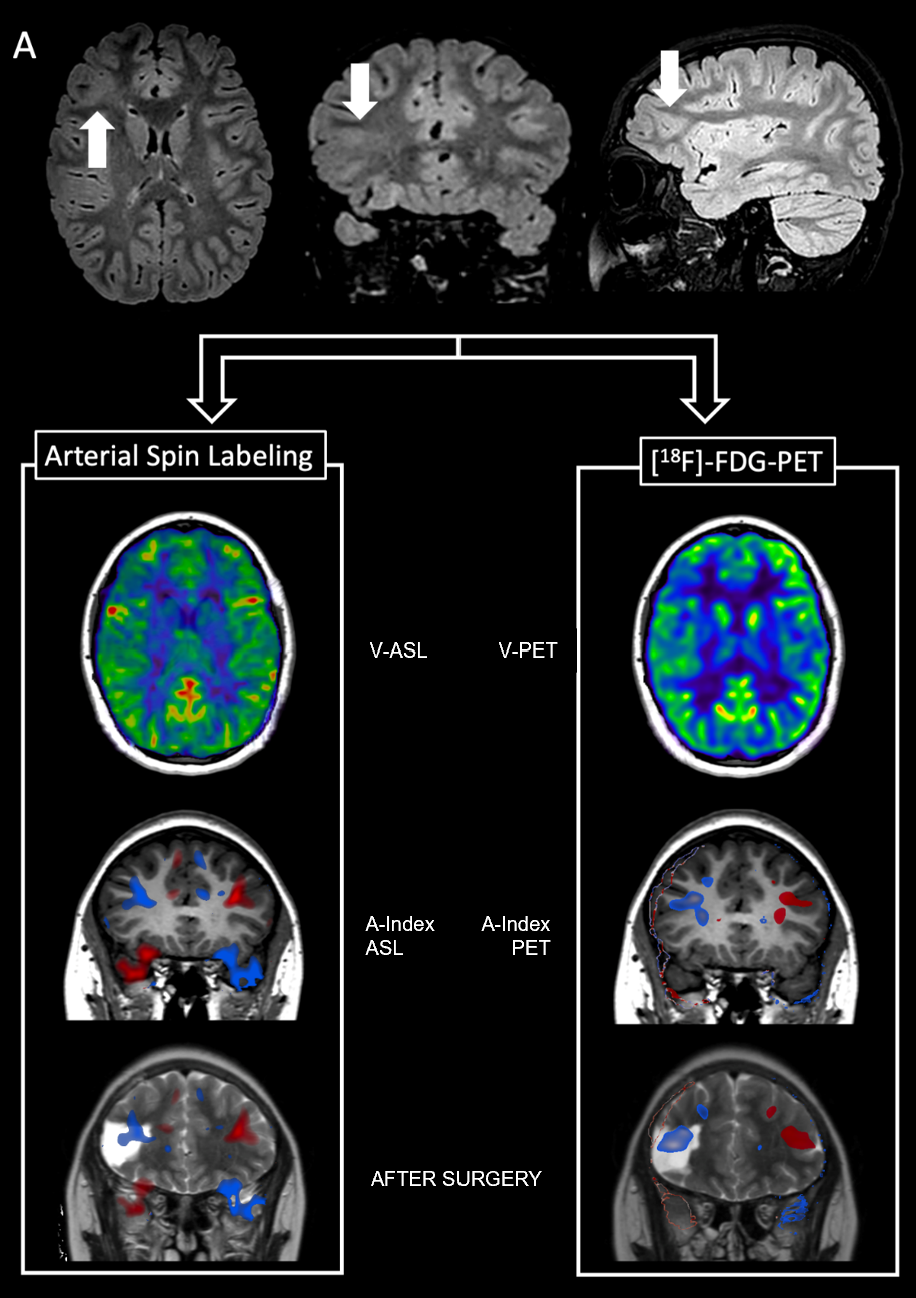
**

**Supplementary Figure 1.** **MR images of Engel class I patient with focal right frontal epilepsy undergoing surgery.**  3D-FLAIR images (A) show right frontal focal cortical dysplasia (white arrows). Qualitative analysis of the ASL-CBF map (V-ASL) revealed faint changes of CBF and [^18^F]FDG-PET map (V-PET) identified a slight reduction of CBF and SUV in the right frontal region. Quantitative voxel-based analysis of the Asymmetry Index of both ASL (A-index-ASL) and PET (A-index-PET) confirmed a region of asymmetry in the frontal lobes with reduced perfusion/metabolism in the right side. Coronal T2-weighted image acquired after surgery shows a resected area in the right frontal lobe. A-index-ASL and A-index-PET maps overlaid on post-surgery T2-weighted image show correspondence of the cluster of significant results with the area of resected lesion. To notice, A-index-ASL and A-index-PET maps overlaid on post-surgery T2-weighted images are showed for visualization only and are not part of formal analysis.

**Supplementary table 1.** Rates of agreement with the epileptic zone (anatomo-electroclinical-correlation) through neuroimaging techniques before and after voxel-based asymmetry index post-processing in the sub-group of patients in which the epileptogenic zone was completely removed and were free from seizures at 12 months follow up. Significant results are reported in **bold**.

|  | *Neuroimaging concordance proportion* | | | | *Comparisons* | | | | | | |
| --- | --- | --- | --- | --- | --- | --- | --- | --- | --- | --- | --- |
|  | *V-ASL* | *A-Index-ASL* | *V-PET* | *A-Index-PET* | V-ASL  vs  *A-Index-*ASL | V-ASL  vs  *A-Index-*PET | V-ASL  vs  V-PET | *A-Index-*ASL vs  V-PET | | *A-Index-*ASL vs  AI-PET | V-PET vs  A-*Index*-PET |
| Agreement |  |  |  |  | 0.115 | 0.198 | 0.096 | 0.801 | 0.675 | | 0.392 |
| - No concordance | 7 (53.85%) | 2 (15.39%) | 1 (7.69%) | 2 (15.39%) |  |  |  |  |  | |  |
| - Partial concordance | 2 (15.39%) | 3 (23.08%) | 4 (30.77%) | 5 (38.46%) |  |  |  |  |  | |  |
| - Complete concordance | 4 (30.77%) | 8 (61.54%) | 8 (61.54%) | 6 (46.15%) |  |  |  |  |  | |  |
| Dichotomized |  |  |  |  | 0.131 | 0.131 | **0.041** | 0.9 | 0.9 | | 0.9 |
| - No concordance | 7 (53.85%) | 2 (15.39%) | 1 (7.69%) | 2 (15.39%) |  |  |  |  |  | |  |
| - Concordance | 6 (46.15%) | 11 (84.62%) | 12 (92.31%) | 11 (84.62%) |  |  |  |  |  | |  |

Legend: A-Index-ASL: arterial spin labelling MRI voxel-based asymmetry index analysis; A-Index-PET: [^18^F]fluorodeoxyglucose PET voxel-based asymmetry index analysis; V-ASL: arterial spin labelling MRI visual analysis; V-PET: [^18^F]fluoro-deoxiglucose-PET visual analysis.

* Mc Nemar-Bowker for marginal homogenety

# Mc Nemar test with continuity correction
